# Dynamical Properties of Plasmon Polaritons in Nanorings Driven by Cassini-Ordered Emitters

**DOI:** 10.3390/nano15080576

**Published:** 2025-04-10

**Authors:** Gennadiy Burlak, Gustavo Medina-Ángel

**Affiliations:** 1 Centro de Investigación en Ingeniería y Ciencias Aplicadas, Universidad Autónoma del Estado de Morelos, Av. Universidad 1001, Cuernavaca 62209, Mexico; 2 Facultad de Contaduría, Administración e Informática, Universidad Autónoma del Estado de Morelos, Av. Universidad 1001, Cuernavaca 62209, Mexico

**Keywords:** plasmon-polaritons, nanorings, cassini-ordered emitters, FDTD

## Abstract

The dynamics of plasmon polaritons (PPs) in a periodic lattice of dispersed nanorings (NRs) with embedded quantum nanoemitters (NEs) arranged according to the Cassini–Bernoulli lemniscate (LB) is studied. The field structure and the dynamics of the NE (quantum polarization) depend significantly on the plasma frequency $\omega_{p}$ of the NR. We show that in the vicinity of the intersection of the LB branches (a region of high emitter density) located in the nanoring gaps, there is a significant enhancement of the optical field intensity and quantum correlations in the emitter subsystem. This effect may allow the coherent amplification of terahertz PPs (studied recently via free-electron-stimulated emission) in a lattice of NRs with the emission of embedded NEs.

## 1. Introduction

The inclusion of dispersed single-walled carbon nanotubes or nanorings (NRs) in the working space of nanoemitters (NEs) considerably changes the properties of the electromagnetic field, whose structure depends significantly on the plasma frequency $\omega_{p}$ of NRs. In such a hybrid system, it becomes possible to control the properties of local optical fields, allowing the creation of miniature low-threshold coherent sources with tunable polarization [1,2]. Nowadays, the investigations of the spectral tunability of localized plasmon resonances in conducting nanostructures (plasmonic nanorings) have attracted considerable spectroscopic studies on a spatial scale of several nanometers in the field [3,4,5,6,7,8]. Such structures allow the exploitation of the enhancement of the electromagnetic field for applications including single-molecule sensing at visible frequencies in near-infrared [9,10,11,12,13], and have stimulated various spectroscopic nanometers [2,14,15,16,17,18,19,20,21,22,23,24]. The energy transfer between light and matter is an important aspect of recent optical surface-wave-mediated studies, and the coherent amplification of THz surface plasmon polaritons via free-electron-stimulated emission has already been demonstrated [25]. Considerable interest is devoted to the study of the linear and nonlinear plasmonic properties of nanohybrids and nanocomposites made of metallic nanoparticles and quantum emitters (QEs) [26,27,28,29,30]. For optical frequencies, in such systems the dynamic properties of electromagnetic waves in plasmonic structures dominate, where the surface PPs mainly contribute. In the real case of lossy NRs with embedded NEs, additional factors become important, however. The plasmonic fields of the dispersing NRs perturb the energy levels of the quantum NEs; thus, the PPs in the NRs will change the internal quantum degrees of freedom of the NEs. Hybrid systems with randomly distributed nanoemitters integrated into a periodic system of carbon nanotubes have recently been studied [31]. However, the uniform distribution of NEs allows the creation of media with simple radiative properties only. In this paper, it is shown that embedding NEs into more complex curves (or 2D surfaces) with irregular or non-smooth behavior may allow the creation of an advanced radiative medium. The Bernoulli lemniscate (LB) is studied, which consists of two symmetric branches intersecting at a central nodal point (LB-node) as a perspective pattern. In the vicinity of the central node, there occurs accumulation of a number of radiating sources, leading to an increase in the optical field density. Due to the nonlinearity of the lasing NEs at such a point, new field dynamics, different from the linear case, can arise.

**Figure 1:**
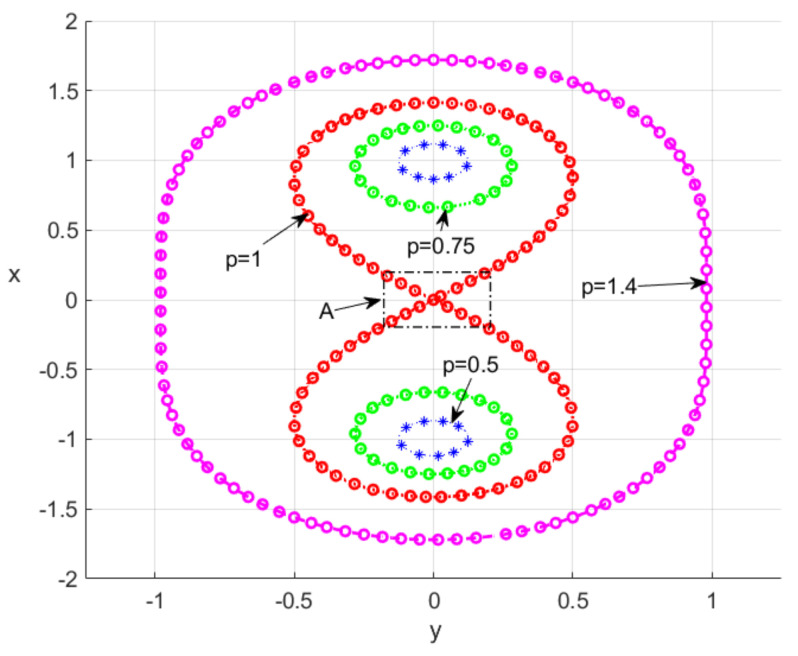
The Cassini oval (CO) is a plane curve defined as a set of points in the plane such that the product of the distances to two fixed points (focuses) is constant. This curve is defined by the equation $\left({\left(x+a\right)}^{2}+y^{2}\right)*\left({\left(x-a\right)}^{2}+y^{2}\right)=b^{4}$. The CO is shown for the cases p=b/a=0.5, 0.75, 1.0, 1.4 (a=1 is used). The arrow *A* exhibits the region with largest density of NEs.

From Figure 1, one can see that the shape of the CO depends significantly on the parameter p=b/a. When p<1, the curve consists of two unconnected loops, each containing a focus, but at p>1, the curve is a single loop spanning both focuses. When p=1, the curve is a Bernoulli lemniscate (LB) with a double point at the origin, which is depicted by symbol A. It can be seen that in this neighborhood, the NE density increases significantly. Therefore, in this paper we mainly concentrate on the LB case.

The rest of this paper is organized as follows. In Section 2, we formulate the basic field equations in the considered hybrid system of NEs connected to NRs. In Section 3, we investigate the nonlinear field related to the laser emission and plasmon-assisted dynamics of NEs coupled to NRs. In Section 4, we study the near-field-to-far-field transformation of the field structures arising from PP resonant coupling. The last sections contain the discussion and our conclusions.

## 2. Basic Equations

It should be noted that the considered 2D periodic system consists of a small number of nanorings (6×6) and does not possess translational symmetry. In such a system, the theory of estimation of eigenfrequencies and the field structure developed for large periodic media is not accurate enough. Therefore, in our paper we apply another approach (see [32]), which allows us to study both longitudinally and transversely polarized dipole modes for conducting cylinders and a dimmer with an arbitrary aspect ratio. In the case of conducting particles, the frequency of longitudinal oscillations tends to the plasma frequency $\omega_{p}$, and the coupling of localized surface plasmons leads to significant hybridization of plasmon excitations already in the case of the dimmer [32]. However, the role of such a hybridization for systems with periodical rings and its relation to the dynamics of quantum emitters remains insufficiently investigated thus far, although it is a logical extension of other works in this area. In this paper, we study the case of a periodic system of dispersing nanorings with incorporated NEs, in which PP interactions with reconnections of optical fields occur. To study the properties of such a hybrid system, we have to deal with time-dependent Maxwell’s equations in the lattice of 2D nanorings, coupled with the rate equations of electron population (within a semiclassical theory) for NEs [33]. However, the field dynamics of such a hybrid system do not have a simple analytical description. Therefore, a well-known FDTD (finite-difference time-domain) [34] approach was used to explore the dynamic properties of the field in such a periodic medium with gain. Maxwell equations for the electric E and magnetic H fields read [35,36]

(1) $$\nabla \times E=-\mu_{0}\frac{\partial H}{\partial t},\;\nabla \times H=\varepsilon_{0}\frac{\partial E}{\partial t}+J+\frac{\partial P}{\partial t},\;\;$$

where $J=\sum_{k}J_{k}\left(R_{k}^{r},t\right)\delta_{{\mathrm{rR}}_{k}^{r}\;}$ is the electrical current of the PP in the NRs placed in spatial positions $R_{k}^{r}$, $P=\sum_{k}P_{k}\left(R_{k}^{s},t\right)\delta_{{\mathrm{rR}}_{k}^{s}}$ is the polarization of electrons in the embedded NEs placed in $R_{k}^{s}$, $\varepsilon_{0}$ and $\mu_{0}$ are the permittivity and permeability of free space, respectively, and $\delta_{{\mathrm{rR}}_{k}^{s}}=1,\;if\left(r=R_{k}^{s}\right)$, otherwise =0, is the Kronecker delta symbol. Here, the sums run over all the NRs ($k=1,N_{r}$) and NEs ($k=1,N_{s}$), respectively. In Equation (1), the electrical current of conducting electrons in the NR obeys the material equation [34] ${\dot{J}}_{k}+\gamma_{e}J_{k}=\varepsilon_{0}\omega_{p}^{2}E$, where $\gamma_{e}$ is the collision frequency of electrons, $\varepsilon_{h}$ is the dielectric constant of the host medium of the NR, and the plasma frequency is $\omega_{p}={\left(4\pi n_{0}e^{2}/m_{e}\right)}^{1/2}$, with the density $n_{0}$ of the electrons having charge −e<0 and mass $m_{e}$. For quasiclassical approximation in the single electron case, the equation for $P_{k}$ in the vicinity of the embedded NEs reads [33]

(2) $$\frac{\partial^{2}P_{k}}{\partial t^{2}}+\Delta \omega_{a}\frac{\partial P_{k}}{\partial t}+\omega_{a}^{2}P_{k}=\frac{6\pi \varepsilon_{0}c^{3}}{\tau_{21}\omega_{a}^{2}}\left(N_{1,k}-N_{2,k}\right)E_{k}.$$

To complete the model, we add the rate equations [33] for the occupation levels of emitters $N_{i,k}=N_{i}\left(R_{k}^{s},t\right)$ (following [35], we consider that the NEs are four-level quantum dots):

(3) $$\frac{\partial N_{0,k}}{\partial t}=-A_{r}N_{0,k}+\frac{N_{1,k}}{\tau_{13}},\;\frac{\partial N_{3,k}}{\partial t}=A_{r}N_{0,k}-\frac{N_{3,k}}{\tau_{02}},$$

(4) $$\frac{\partial N_{1,k}}{\partial t}=\frac{N_{2,k}}{\tau_{32}}-M_{k}-\frac{N_{1,k}}{\tau_{13}},\;\frac{\partial N_{2,k}}{\partial t}=\frac{N_{1,k}}{\tau_{12}}+M_{k}-\frac{N_{2,k}}{\tau_{02}},$$

(5) $$M_{k}=\frac{{\left(i_{\mathrm{ph}}\cdot E\right)}_{k}}{\hbar \omega_{a}},\;i_{\mathrm{ph}}k=\frac{\partial P_{k}}{\partial t}.$$

Here, $\Delta \omega_{a}=\tau_{21}^{-1}+2T_{2}^{-1}$, where $T_{2}$ is the mean time between dephasing events, $\tau_{21}$ is the decay time from the second atomic level to the first one, $\omega_{a}$ is the frequency of radiation (see, e.g., [33]), the lifetimes of upper and lower lasing levels are $\tau_{21}$ and $\tau_{10}$m, respectively, and $M_{k}$ is the induced emission rate or excitation rate, depending on its sign [35]. Coefficient $A_{r}$ is a certain pumping rate from the ground level ($N_{0}$) to the third level ($N_{3}$), which is proportional to the pumping intensity in experiments [35]. The normalized electron density $N_{0}+N_{1}+N_{2}+N_{3}=1$ and the pump rate $A_{r}$ are the controlled variables, according to [35,37].

**Figure 2:**
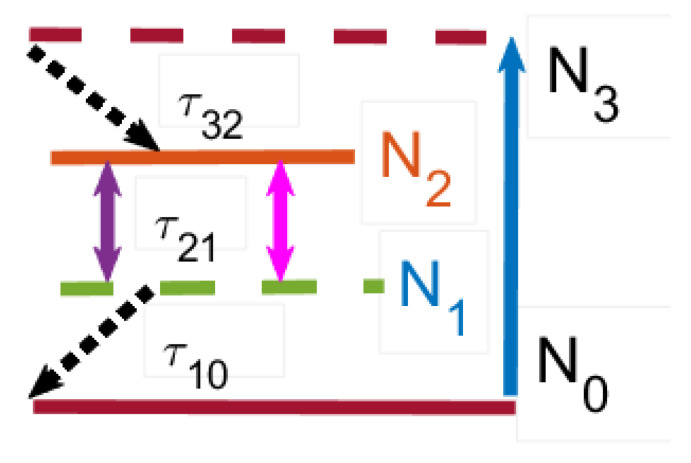
Schematic representation of an NE as a four-level system, see Equations (3)–(5). An external mechanism pumps electrons from the ground level ($N_{0}$) to the third level ($N_{3}$) at a certain pumping rate. After a short lifetime $\tau_{32}$, electrons can nonradiatively transfer to the second level ($N_{2}$). The second level ($N_{2}$) and the first level ($N_{1}$) are called the upper and the lower lasing levels. Electrons can be transferred from the upper to the lower level by both spontaneous and stimulated emission. At last, electrons can nonradiatively transfer from the first level ($N_{1}$) back to the ground level ($N_{0}$) [33].

**Figure 3:**
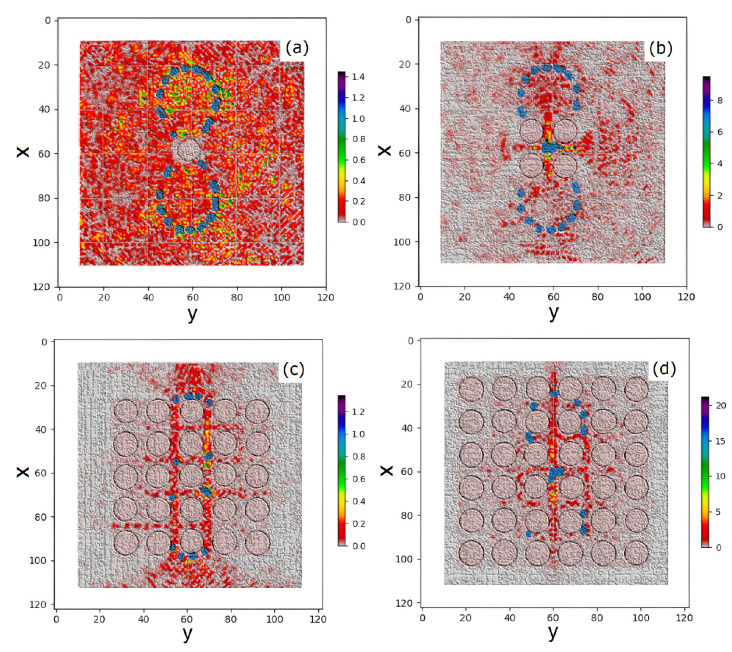
The FDTD simulations of the field dynamics in the periodic structure (at time *t*∼10) for the optical field $E_{z}$ component (the red, arbitrary units) at $\omega_{p}=2.3$ THz and different numbers of nanorings (NRs) (black circles) in the lattice with the embedded nanoemitters (NEs) (blue points). NEs are embedded according to the shape of the Bernoulli lemniscate (LB): (**a**) NR=1, (**b**) NR=4(2×2), (**c**) NR=25(5×5), and (**d**) NR=36(6×6).

Figure 2 exhibits the schematic representation of an NE as a four-level system, see Equations (3)–(5). An external mechanism pumps electrons from the ground level ($N_{0}$) to third level ($N_{3}$) at a certain pumping rate. After a short lifetime $\tau_{32}$, electrons can nonradiatively transfer to the second level ($N_{2}$). The second level ($N_{2}$) and the first level ($N_{1}$) are called the upper and the lower lasing levels. Electrons can be transferred from the upper to the lower level by both spontaneous and stimulated emission. At last, electrons can nonradiatively transfer from the first level ($N_{1}$) back to the ground level ($N_{0}$). We consider the system (1)–(4), which combines Maxwell’s equations containing the PP field with semiclassical optical emission from laser NEs. In our simulations, we consider a gain medium with parameters close to GaN powder, see Refs. [35,37]. In Equations (2)–(4), the frequency $\omega_{a}$ is $2\pi \;\times \;3\;\times \;10^{13}\;\mathrm{Hz}$, the lifetimes are $\tau_{32}=0.3\;\mathrm{ps}$, $\tau_{10}=1.6\;\mathrm{ps}$, $\tau_{21}=16.6\;\mathrm{ps}$, and the dephasing time is $T_{2}=0.218\;\mathrm{ps}$. In our simulations, we use the dimensionless time *t* re-normalized as $t\to tc/l_{0}$, where $l_{0}=10$ μm is the typical spatial scale and *c* is the light velocity in the vacuum. (For completeness, we point out that such a time scale corresponds to the dimension time scale 0.1ps). The considered model couples the population rate equations at different levels of NEs with field equations of the PP in the vicinity of the NR lattice. Therefore, the optical emission of the embedded emitters is affected by the plasmon polariton excitation in the rings, which leads to complex optical field dynamics. The system (Equations (1)–(4)) is essentially nonlinear due to the products $\left(N_{1,k}-N_{2,k}\right)E_{k}$ on the right hand of the polarization Equation (2) and ${\left(i_{\mathrm{ph}}\cdot E\right)}_{k}$ in the rate level populations (Equation (5)). This significantly complicates the study of the field dynamics in the considered 2D setup. The system under consideration consists of a periodic lattice of conducting NRs (black circles) and embedded NEs (blue dots) emitting an optical field (the red), see Figure 3. Such NEs (ordered according to LB shape) are embedded into a conducting NR lattice, where the PPs are excited. Figure 3 shows a sketch of our FDTD simulations at initial times *t*∼10 for the cases of a lattice with different numbers of NRs, (a) NR=1, (b) NR=4(2×2), (c) NR=25(5×5), and (d) NR=36(6×6). One can see that with an odd number of NRs [cases (a) and (c)], a single NR occupies the center part of the lattice, where the intersection point of the LB branches is located, so in the vicinity of this point the number (density) of NEs is small. With an even number of NRs, (b) and (d), the intersection point of the LB branches lies in the gap of the NR lattice, so in the vicinity of this point the NE density can be large.

## 3. Lasing of Nanoemitters in the System with Nanorings

**Figure 4:**
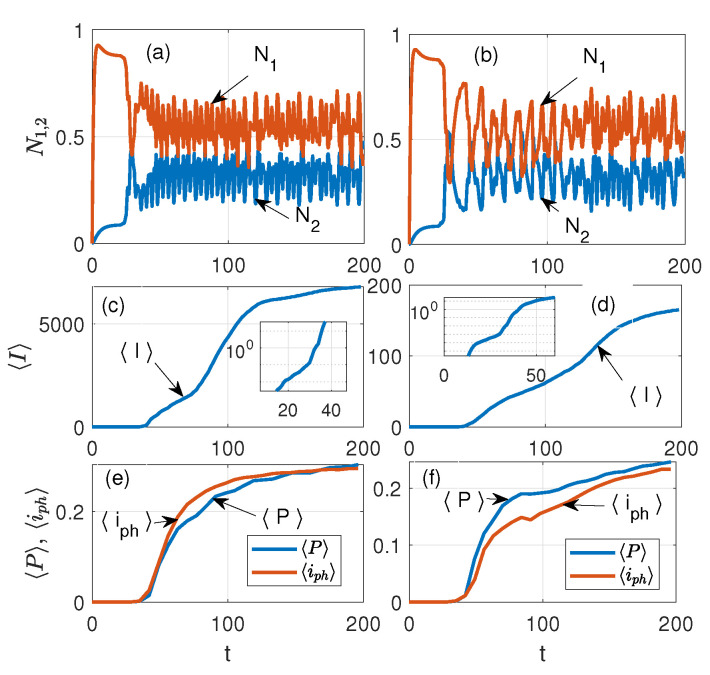
Temporal dynamics of FDTD simulations for the system Equations (1)–(5) at different values of the plasma frequency of NRs: on the left $\omega_{p}=2.3$ THz, and on the right, $\omega_{p}=2.3\times 10^{-2}$ THz, receptively, for the case of a 5×5 lattice: (**a**,**b**)—the laser levels $N_{1},N_{2}$ in the NE, see Equation (4); (**c**,**d**)—the average current in the nanorings $\langle J\rangle$, see Equation (6); and, (**e**,**f**)—the average amplitudes of the polarization |P| and photocurrent $i_{ph}=\partial |P|/\partial t$ in the nanoemitters, respectively. The insets in (**c**,**d**) show the dynamics of $\langle J\rangle$ in the log scale. We notice a drastic difference in the amplitudes $\langle J\rangle$ in (**c**,**d**) already at t∼100. In (**c**–**f**), arbitrary units are used.

In this section, we study the dynamics of the system (1)–(4), which combines Maxwell’s equations for the PP field with semiclassical optical field emission from the laser emitters, displayed in Figure 3. To study the optical field structure in the NR lattice with a gain-assisted embedded NE, we use the well-known FDTD technique [34]. We use an advanced technique, where the standard FDTD approach is extended by calculating the dynamics of a quasiclassical system consisting of polarization (Equation (2)) related to the population dynamics in the four-level laser emitters (Equations (3) and (4)) at each time step, see more details in Ref. [31]. In our FDTD simulations, we consider a 2D lattice of NRs placed in the computing domain L×L (we used various L=100,132,142,242). The standard PML boundary conditions on the boundaries of the FDTD grid are applied to avoid the reflection of electromagnetic waves at the boundary interfaces [34,38]. In the used scale, the typical size of nanoemitters is orders smaller with respect to NRs’ typical size; therefore, the emitters are simulated by point-like sources. As it turns out, the dynamics of such a system depend drastically on the plasma frequency of the dispersive nanorings in the terahertz band. Therefore, in the following we will consider two cases in more detail, when $\omega_{p}=2.3$ THz and $\omega_{p}=2.3\times 10^{-2}$ THz. In our simulation, we deal with the general case of 3D vector electromagnetic E and H fields. But, as the simulations show, in such a system the TM electromagnetic waves $\left[E_{z},H_{x},H_{y}\right]$ are mainly generated. Such an observation is supported by the theory in Ref. [32], where it was shown that in the case of nano-objects in the disk limit (h/r≪1, where *r* and *h* are the radius and height, respectively) the main contribution gives an $E_{z}$ ($E_{\|}$)-longitude field, while the transverse field contributes at h/r∼1 [32]. Thus, in the disk limit (h/r≪1) one can obtain $\omega_{0,\|}≃\omega_{p}$, which approaches the plasma frequency $\omega_{p}$ in the limit (h/r→0). For case of TE mode, the corresponding frequency has order $\omega_{0,⊥}/\omega_{p}∼\sqrt{h/r}$, which is small in our case (h/r→0). For simplicity in Figure 1, Figure 2, Figure 3 and Figure 4, only $|E_{z}|$ field components are displayed.

Figure 4 exhibits the temporal dynamics of FDTD simulations for Equations (1)–(5) at different values of the plasma frequency in the case of a 5×5 lattice: (a) and (b) show the laser levels $N_{1},N_{2}$ in the NE, see Equation (4); (c) and (d) are the average current in the nanorings $\langle J\rangle$, see Equation (6); and (e) and (f) are the average amplitudes of the polarization |P| and photocurrent $i_{p}=\partial |P|/\partial t$ in the quantum nanoemitters, respectively (arbitrary units are used). The plasma frequency of the PP on the left of Figure 4 is $\omega_{p}=2.3$ THz, and on the right, $\omega_{p}=2.3\times 10^{-2}$ THz, respectively. [The dynamics of $N_{0,3}$ levels (Equation (3)) are not displayed in (a) or (b)]. We notice a drastic difference in the amplitudes $\langle J\rangle$ in (c) and (d) for different values of $\omega_{p}$ of the NRs already at *t*∼100.

**Figure 5:**
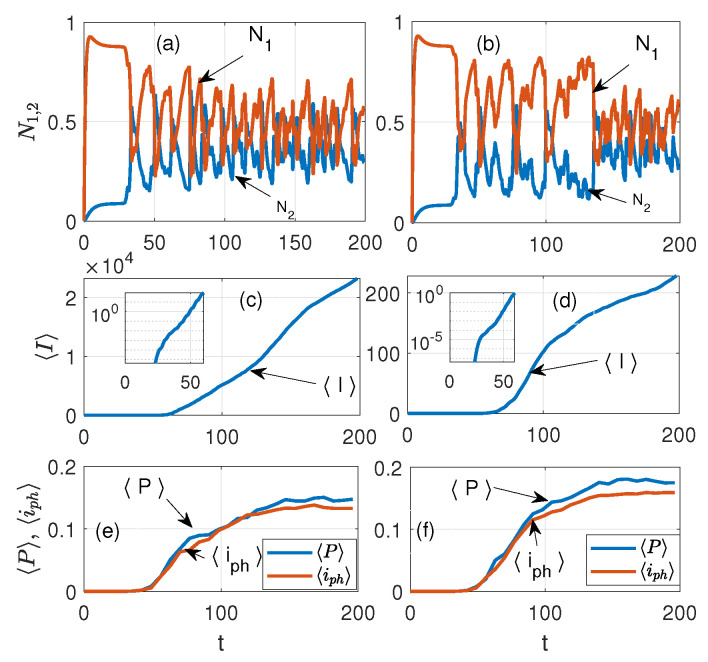
The same as Figure 4 but for a 6×6 lattice, the letters (**a**–**f**) indicate same as in Figure 4.

**Figure 6:**
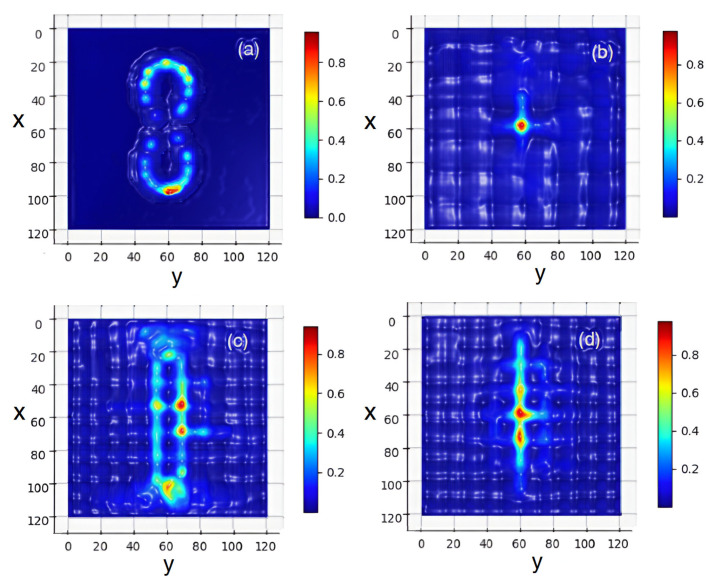
Spatial structure of the normalized average field energy in the far zone (arbitrary units) at long times (t≥100), and $\omega_{p}=2.3$ THz for different numbers of NRs in the lattice with embedded NEs (cf. Figure 3) at (**a**) NR=1, (**b**) NR=4(2×2), (**c**) NR=25(5×5), and (**d**) NR=36(6×6).

At $\omega_{p}=2.3$ THz, the amplitude of $\langle J\rangle$ is about 30 times larger with respect to $\langle J\rangle$ for $\omega_{p}=0.023$ THz. The insets in (c) and (d) show the dynamic of $\langle J\rangle$ in the log scale, which allows us to see the details of $\langle J\rangle$ generation at initial times. From the latter, one can see that the generation of $\langle J\rangle$ coincides with the onset of lasing in the nanoemitters and the transition of the system into the nonlinear regime, see Figure 4a,b. Figure 5 shows the same as Figure 4 but for a 6×6 lattice. One can see that for the 6×6 case, the difference in the amplitudes $\langle J\rangle$ in (c) and (d) for different $\omega_{p}$ frequencies is approximately 50 times greater. From Figure 6, one can observe that with time the dynamics of the field acquire a more ordered shape, which weakly dependents on the details of the NE distribution. As Figure 6 shows, at t>25 the energy exchange between the NE and the PP occurs mainly in the gaps of the NR lattice. The latter indicates the nonlinear dynamics (laser generation) of such an interaction in the subsystem of the NE. Since the dynamics of the PP in the NR subsystem strongly depend on the plasma frequency $\omega_{p}$ of the PP, we should expect that the dynamics of coupled emitters will depend on the $\omega_{p}$ too. Therefore, to understand the dynamics in such a hybrid environment, it is useful to study the properties of the average current in the NR lattice, which reads as

(6) $$\langle J\rangle ={\langle J(\omega_{p})\rangle }_{N}={\left(N\right)}^{-1}{\left(\sum_{N}{\left|I_{i,j}\right|}^{2}\right)}^{0.5},$$

where $I_{i,j}$ is the current in the lattice of the i,j nanoring, and *N* is the number of NRs in the x,y directions. Corresponding dynamics for different lattices are displayed in Figure 4 and Figure 5, where the time dependencies of populations of lasing levels $N_{1,2}$ of NEs (see Equation (3)) and the average current $\langle J\rangle$ in the lattice of nanorings are shown. From Figure 4, it can be seen that at t≈25 there is a rapid growth in $\langle J\rangle$ when lasing is observed in the nanorings. The latter indicates the onset of NE coupling with the PP in the NR. It is instructive to investigate the spatial distribution of the energy density in such a system. Figure 6 shows such a normalized distribution of the average field energy from the long-time simulations, and $\omega_{p}=2.3$ THz for different numbers of NRs in the lattice with embedded NEs (cf. Figure 3): (a) NR=1, (b) NR=4(2×2), (c) NR=25(5×5), and (d) NR=36(6×6). In Figure 6a, the single NR occupies the center of the lattice; thus, the optical field structure is close to the general LB shape, but without the intersection point of the LB branches. Therefore, the field amplitude of the NE decreases in the vicinity of this region. Panel (c) of [5 × 5] shows a significant interference of the PP fields in the gaps of periodic NRs, which leads to field ordering along the symmetry axis of the LB. One can see the high energy density and amplitude (PP condensation) in the case of the 6×6 lattice, Figure 3d. With an even number of nanorings in the lattice (see panels (b) and (d)), the center of the LB is located inside the NR lattice gap, so the NE density here is significant; the latter leads to the appearance of field peaks along the x−axis of the LB, see Figure 6d.

## 4. Near-Field-to-Far-Field Transformation

For possible experimental investigation of the structure of the optical fields displayed in Figure 6 in the near zone, it is of significant interest to analyze the field structure generated by radiated nanoemitters in the far zone. To obtain the far-field radiation pattern, we calculate the field vectors that are sufficiently far (r≫λ, λ is the wavelength) from the light emitters. Following Ref. [39], we separate the calculation domain by a horizontal plane located just above the NR plane. The upper domain is bound by an infinite hemisphere. All field components (*E* and *H*) are assumed to fall off as 1/r, typical of radiation fields. The far-field region is defined as the region of the field where the angular field distribution is essentially independent of the distance from the system. For the system dimension *D*, the far-field region is taken at distances greater than $2D^{2}/\lambda$ from the NR plane.

**Figure 7:**
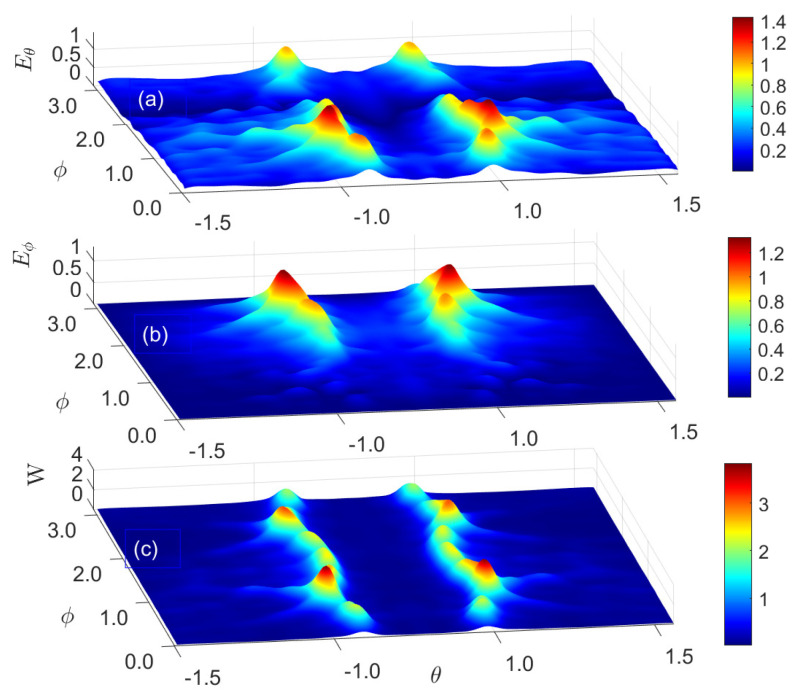
Calculated structure of radiating electrical (from the NR plane) field (arbitrary units) in the far zone with the tangential field components (**a**) $E_{\theta }$ and (**b**) $E_{\phi }$, and (**c**) the average field energy *W*, see Figure 6.

In this region, the field components are essentially transverse, and the angular distribution is independent of the radial distance where the measurements are made. The radiation intensity is related to the far-zone electric field as $W\left(\theta ,\phi \right)=r^{2}/{2\eta |E\left(r,\theta ,\phi \right)|}^{2}≃r^{2}/2\eta [|E_{\theta }{\left(r,\theta ,\phi \right)|}^{2}+|E_{\phi }\left(r,\theta ,\phi \right){|}^{2}],$ where $E_{\theta }$, $E_{\phi }$ are the far-zone electric field components. In the far-field region, only the $E_{\theta }$ and $E_{\phi }$ components of the electric and magnetic fields are dominant, and the E-field reduces to

(7) $$E\left(r,\theta ,\phi \right)≃ike^{-ikr}/2\pi r\left[{\hat{a}}_{\theta }\left(f_{x}\cos\phi +f_{y}\sin\phi \right)+{\hat{a}}_{\phi }\cos\theta \left(-f_{x}\sin\varphi +f_{y}\cos\varphi \right)\right],$$

where the analytical form of coefficients $f_{x,y}$ and the average field energy W(θ,ϕ) are quite long and can be found in Ref. [39]. Here, the position of fields and energy on the sphere is identified using the standard spherical coordinate system θ(0≤θ≤π),φ(0≤φ≤2π), where $k=\omega /c,\eta ={\left(\mu_{0}/\varepsilon_{0}\right)}^{0.5}$ is the impedance of free space, $i={\left(-1\right)}^{0.5}$, and ${\hat{a}}_{\theta },{\hat{a}}_{\varphi }$ are the angular unit vectors corresponding to the spherical angles θ,φ, respectively. Figure 7 shows the calculated structure of the radiating electrical field in the far zone for (a)$E_{\theta }$ and (b)$E_{\phi }$, and (c) is the time-average Poynting vector (average power density) $W=\left(1/2\right)ℜ\left[E\times H^{*}\right]$, and the asterisk means complex conjugate, see Figure 6c. From Figure 7, we observe that the angular structure of the $E_{\theta }$ and $E_{\phi }$ components is inhomogeneous: $E_{\theta }$ is small at ϕ≈2 and $E_{\phi }$ is small for small |ϕ|≪1. The latter leads to angular modulation of the sharp cone edge distribution of the field energy at $\theta =\theta_{0}\approx \pm 0.5$, see Figure 7c. In a far-zone cone, the angle $\theta_{0}$ depends on the periodical structure of the nanoemitter’s field in the near zone.

## 5. Discussion

We explored a hybrid medium consisting of a periodic lattice of dispersed nanorings (free of sources) and a system of quantum emitters distributed in a lemniscate shape, which are embedded in the lattice. It is shown that such a medium has different field behavior depending on the position of the LB node relative to the NR lattice. If the LB node is located outside the lattice (the emitters are in the gap between the nanorings), the emitter fields will lead to a strong coupling of the associated plasmon polariton field in the rings with quantum polarization of the laser system. Such a coupling can stimulate the PP condensation in NRs near the LB center. However, if the location of the LB node is inside the nanorings, such an effect will be blocked by the periodic lattice. Such factors can allow resonant modifications in the field shape due to the coupling of PP excitations in the NR. We observe from Figure 1 and Figure 3 that the density of the NR is greatest near the intersection points of the LB branches. Thus, the situation when a part of the NR is placed in the center in Figure 3a,c) is very different from that in Figure 3b,d. Our calculations confirm that PPs with frequency $\omega_{p}$ are generated in the NR lattice due to the coupling of PPs to the NE through the common optical field. The latter leads to the perturbation of the dynamics of the quantum system of the NE by the field of the PP and to the dependence of the quantum polarization *P* on the value of the plasma frequency $\omega_{p}$ of the PP in HE. Figure 4 and Figure 5 exhibit such an effect. The latter also clarifies why the coupling significantly increases the average current in the NRs. As our modeling shows, such dynamics appear in the essentially nonlinear regime when laser generation occurs in the NE.

## 6. Conclusions

We have studied the dynamical properties of PPs in a periodic lattice of dispersed NRs with incorporated quantum NEs arranged according to the Cassini–Bernoulli (LB) lemniscate. The behavior of the wave spectrum is similar to the phenomenon of surface lattice resonances. However, in our case with very narrow rings, we cannot directly capture the corresponding field structure. On the other hand, as was already mentioned, in the studied hybrid system the TM electromagnetic waves mainly propagate in the lattice, which excite the surface lattice resonances. The latter (indirectly) confirms the dynamics of the surface lattice resonances. The considered effect of laser interaction of amplifying nanoemitters with lattice plasmons precisely placed in a two-dimensional array of NRs in a dielectric medium can be used in optics and also in various photonics structures that support PPs. In such systems, periodic dielectric structures can be integrated with a planar waveguide to create photonic band-edge modes for two-dimensional photonic-crystal lasers (see, e.g., Ref. [40]). The connection between the PP and HE through the common optical field leads to the fact that the field structure and dynamics of HE (quantum polarization) become dependent on the properties of the PP (plasma frequency) in the NR. Significant enhancement of the field amplitude and laser correlations of NEs occur if the crossing point of the LB branches (the region of high LE density increase) is located inside the gaps of the NR lattice.

## Data Availability

The data that support the findings of this study are available from the corresponding author upon reasonable request.
